# Supplementary material for: Low temperature deposition of functional thin films on insulating substrates enabled by selective ion acceleration using synchronized floating potential HiPIMS
Source: Nat Commun. 2025 May 21;16:4719. doi: 10.1038/s41467-025-59911-y (PMC12095684; doi:10.1038/s41467-025-59911-y)
Supplement: Supplementary file 1 — Supplementary Information [file 41467_2025_59911_MOESM1_ESM.pdf]

Supporting Information for:

Low Temperature Deposition of Functional Thin Films on Insulating Substrates Enabled by Selective Ion Acceleration using Synchronized Floating Potential HiPIMS

Jyotish Patidar[1], Oleksandr Pshyk[1], Kerstin Thorwarth[1], Lars Sommerhäuser[1], Sebastian Siol[1*]

[1] Empa, Swiss Federal Laboratories for Materials Science and Technology, Dübendorf, Switzerland

** Corresponding author:*

Sebastian Siol, Sebastian.Siol@empa.ch

ToF-measurement on the Al-pulse

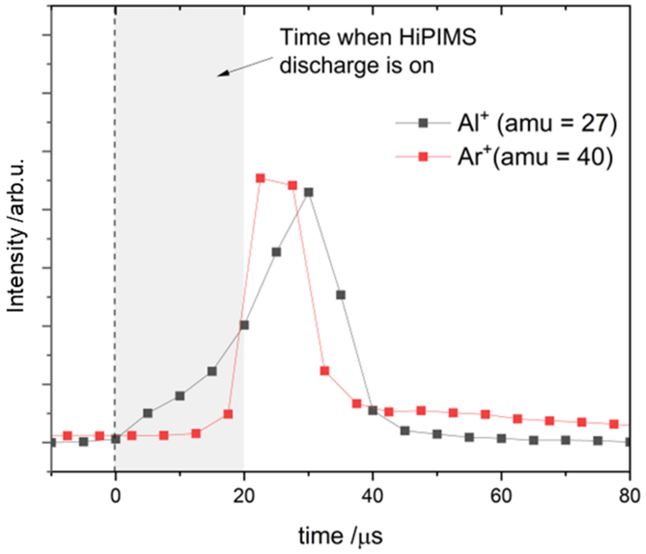

Supplementary Figure 1: Ion flux vs time for of Al and Ar ions originating from the 20 µs HiPIMS pulse of the Al magnetrons.

Measurement of the floating potential

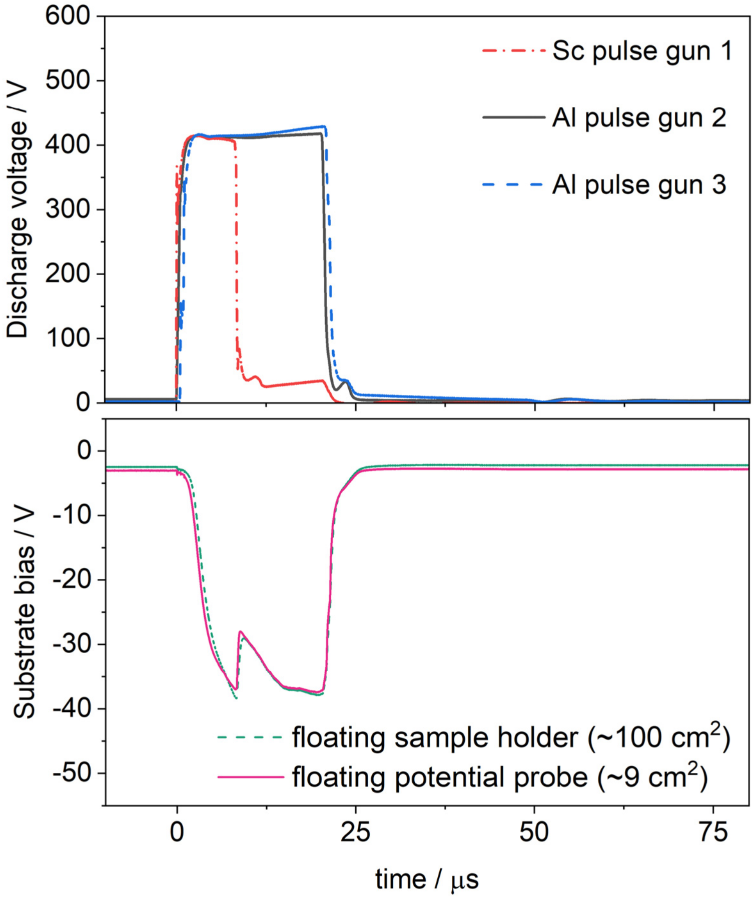

Supplementary Figure 2: Measurement of floating potential on both the substrate holder as well as a custom floating potential probe. The measurement of the floating potential of the substrate holder yields almost identical results to measurements performed using our custom floating potential probe. This floating potential probe is home-built and features an area of 9 cm^2. It is moved to the center of the chamber at the height of the substrate holder. The wire to the probe is insulated, and it is not in electrical contact with any chamber parts. For both the substrate holder and the floating potential probe the measurements are performed using a passive probe with 10 MΩ impedance (Tektronix TPP0502).

Tunability of the floating potential

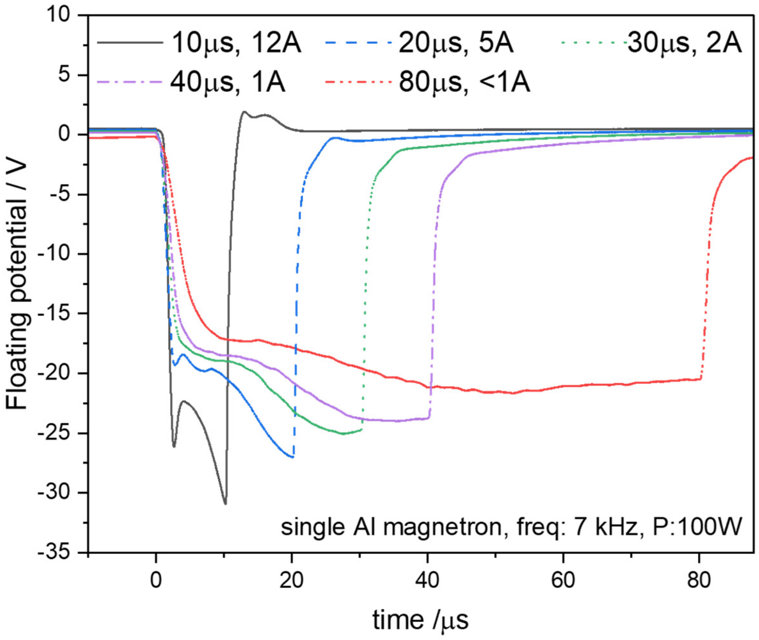

Supplementary Figure 3: Variation of the floating potential as a function of pulse width (at constant average power and frequency), demonstrating its dependence on the discharge voltage. In addition, it is possible to tune the floating potential using other parameters such as the balancing of the magnetron.

Stability of the substrate RF plasma under different process conditions

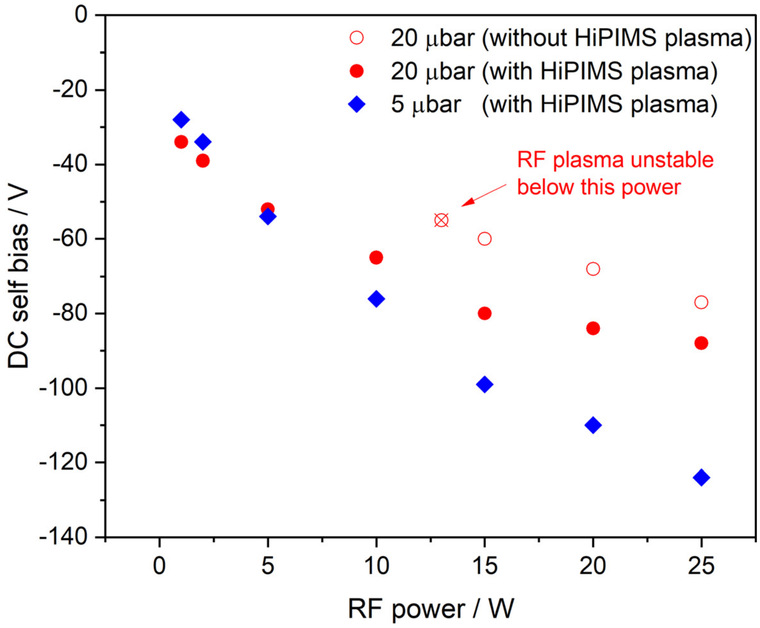

Supplementary Figure 4: Variation of DC self-bias on the substrate holder with RF power, shown for different sputtering pressures, with and without the presence of a HiPIMS discharge. The measurement is performed using a passive probe with 10 MΩ impedance (Tektronix TPP0502) connected to the substrate holder. The reported DC self bias is the time-averaged value. In addition, it was tested at which conditions the RF discharge could be ignited without raising the power or process pressure monetarily. Under these conditions we find, that the minimum attainable DC self-bias at p = 20 µbar is around -50 V. The presence of a HiPIMS plasma stabilizes the RF discharge enabling self-bias voltages as low as -30 V at process pressures of p = 5 µbar.

RF substrate bias potentials used for the reference depositions

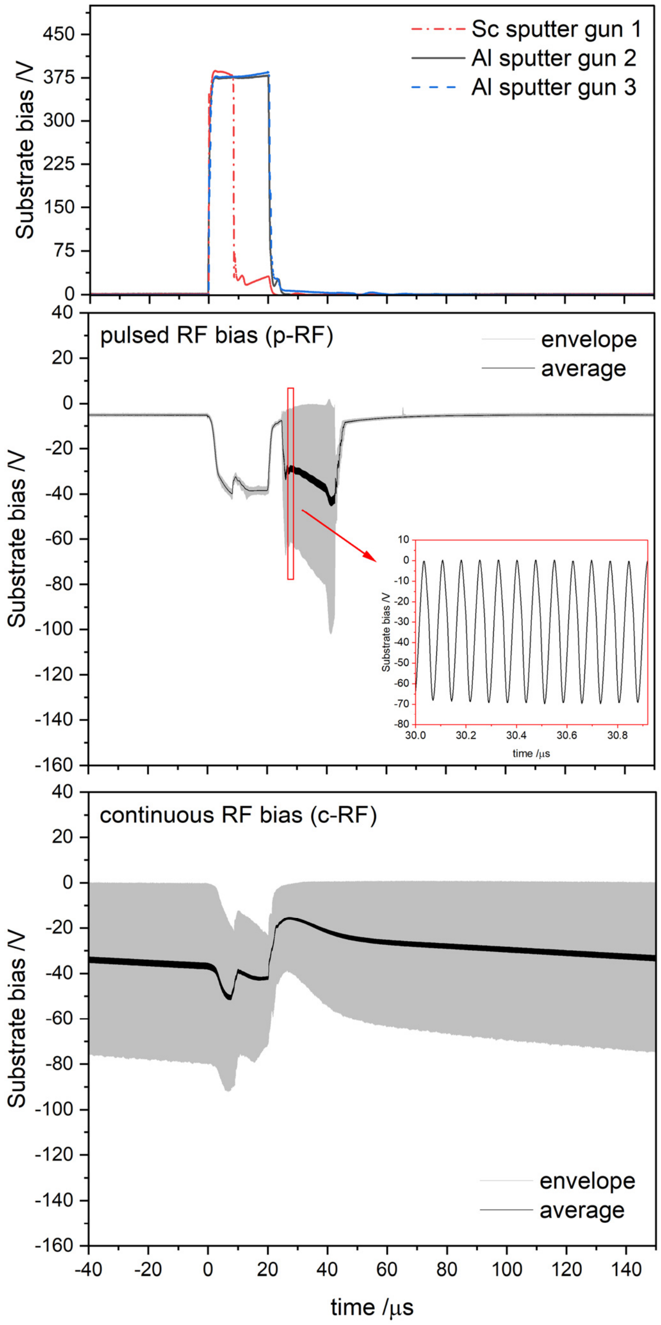

Supplementary Figure 5: Substrate bias potential for the reported depositions using RF substrate biasing. The measurement is performed using a passive probe with 10 MΩ impedance (Tektronix TPP0502) connected to the substrate holder. Shown are the pulsed RF bias synchronized with the Sc ion arrival at the substrate as well as the continuous RF bias. The blackline illustrates the average substrate bias potential, whereas the grey area shows the envelope of the applied RF signal.

Process gas incorporation

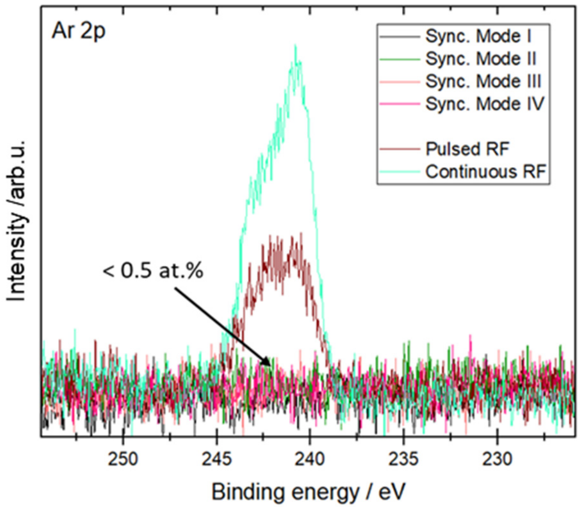

Supplementary Figure 6: Ar 2p core level spectrum from X-ray photoelectron spectroscopy for films deposited using different substrate biasing modes. The Conventional HiPIMS (i.e. no intentional ion acceleration) and SFP-HiPIMS processes (i.e. metal-ion acceleration) show now measurable Ar signal, which is in line with the low residual stress values for those films. In contrast, the RF substrate bias deposition leads to significant Ar-ion incorporation.

X-ray photoelectron spectroscopy (XPS) measurements were performed using a Physical Electronics Quantera spectrometer equipped with a monochromated Al-Ka source. The base pressure during the spectra acquisition is below 10^{-6} Pa. Charge neutralization was achieved using a low-energy electron flood gun, but no Ar-ion neutralization. The photoelectron detection angle was set to 90° to maximize the probing depth. Absolute quantification of the Ar content is difficult due to the formation of surface oxides. The conventional HiPIMS samples, as well as the SFP-HiPIMS samples show no measurable Ar signal, even after long integration times. This corresponds to an Ar content well below the detection limit (< 0.5 at.%).[1] RF biasing on the other hand leads to significant Ar incorporation, which is in line with the high compressive stress measured in these films.

To better quantify the Ar content in the films, Rutherford backscattering spectrometry (RBS) and elastic recoil detection analysis (ERDA) were performed at the 1.7 MV Tandetron accelerator facility of the Laboratory of Ion Beam Physics at ETH Zurich. The RBS measurements were performed using a 2 MeV ^{4}He beam. For the ERDA analysis, a 13 MeV ^{127}I beam was used. **Table S1** shows the bulk Ar-content of the different films. Whereas **Figure S7** shows the RBS/ERDA results for the SFP-HiPIMS as well as pulsed-RF depositions. RBS/ERDA analyses confirm the XPS results and show no measurable Ar incorporation for Sync. Modes I-IV, but significant Ar incorporation for both pulsed-RF and continuous RF substrate biasing. As expected, the highest amount is recorded for the continuous RF bias.

Supplementary Table 1: Ar content measured by RBS/ERDA for different biasing modes

	Sync. Mode I	Sync. Mode II	Sync. Mode III	Sync. Mode IV	Pulsed RF	Continuous RF
**Ar content (at.%)**	0*	0*	0*	0*	0.86	1.22

**below detection limit*

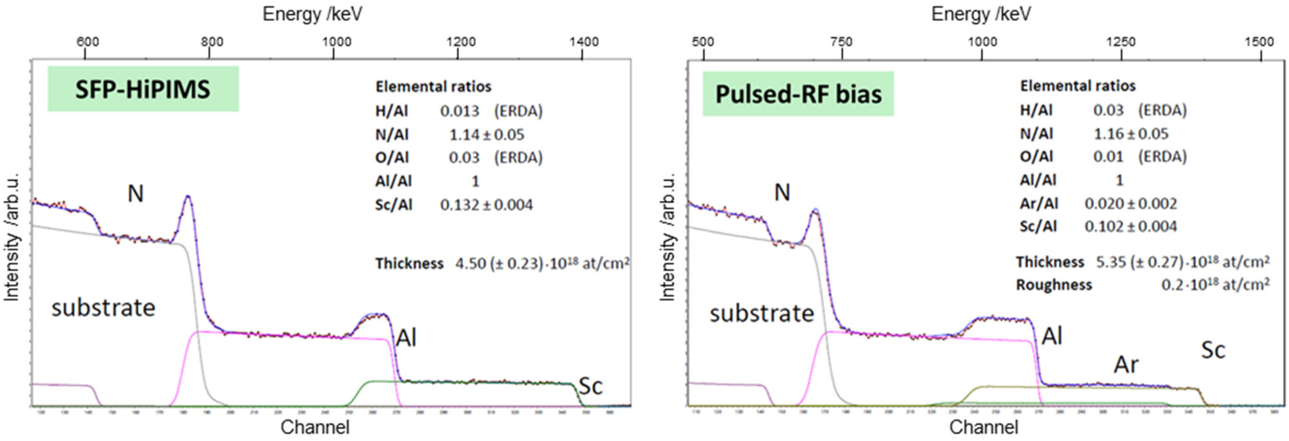

Supplementary Figure 7: Combined RBS/ERDA analysis on AlScN samples deposited using either SFP-HiPIMS or the corresponding pulsed RF substrate biasing. No Ar can be detected in the SFP-HiPIMS film, whereas the pulsed RF deposition leads to significant Ar impurities.

Time averaged Ar ion energy measurements

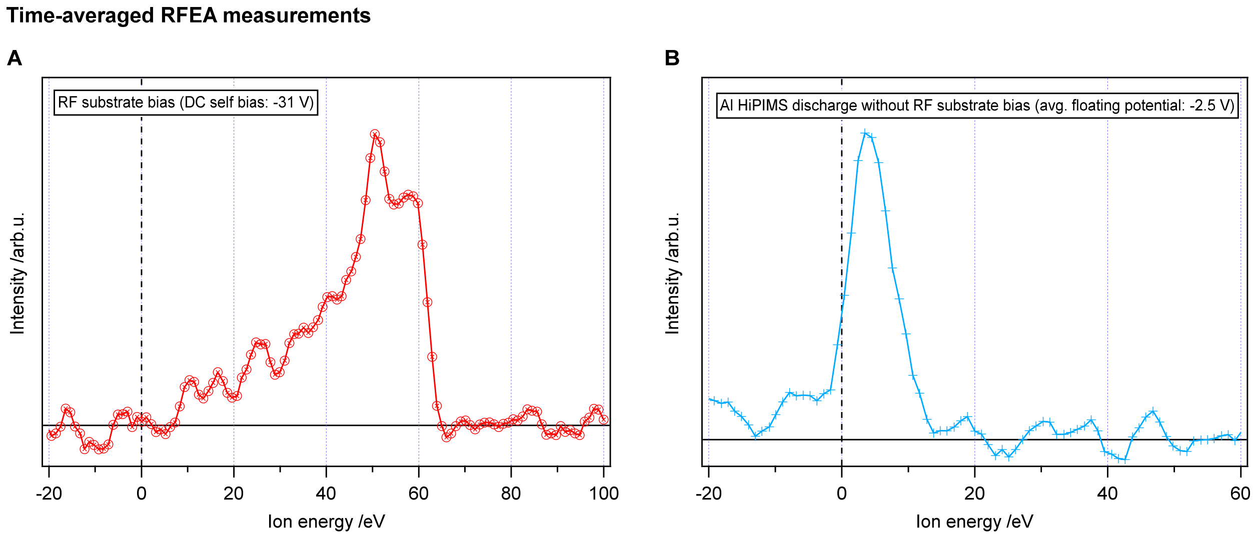

Supplementary Figure 8: Time-averaged retarding field energy analyzer (RFEA) measurements showing the ion energy distribution on the substrate: (a) with RF bias generating a -31 V DC self-bias, and (b) Al HiPIMS discharge with average floating potential of about -2.5 V. The RF substrate bias results in Ar^+-ion energies of over 60 eV.

The RFEA measurements were conducted using an Impedans Semion RFEA button probe in electrical contact with the substrate holder. The measurements were performed using just the RF substrate bias or a single Al HiPIMS discharge with a floating substrate potential (no RF power supply connected).

Surface morphology of deposited films

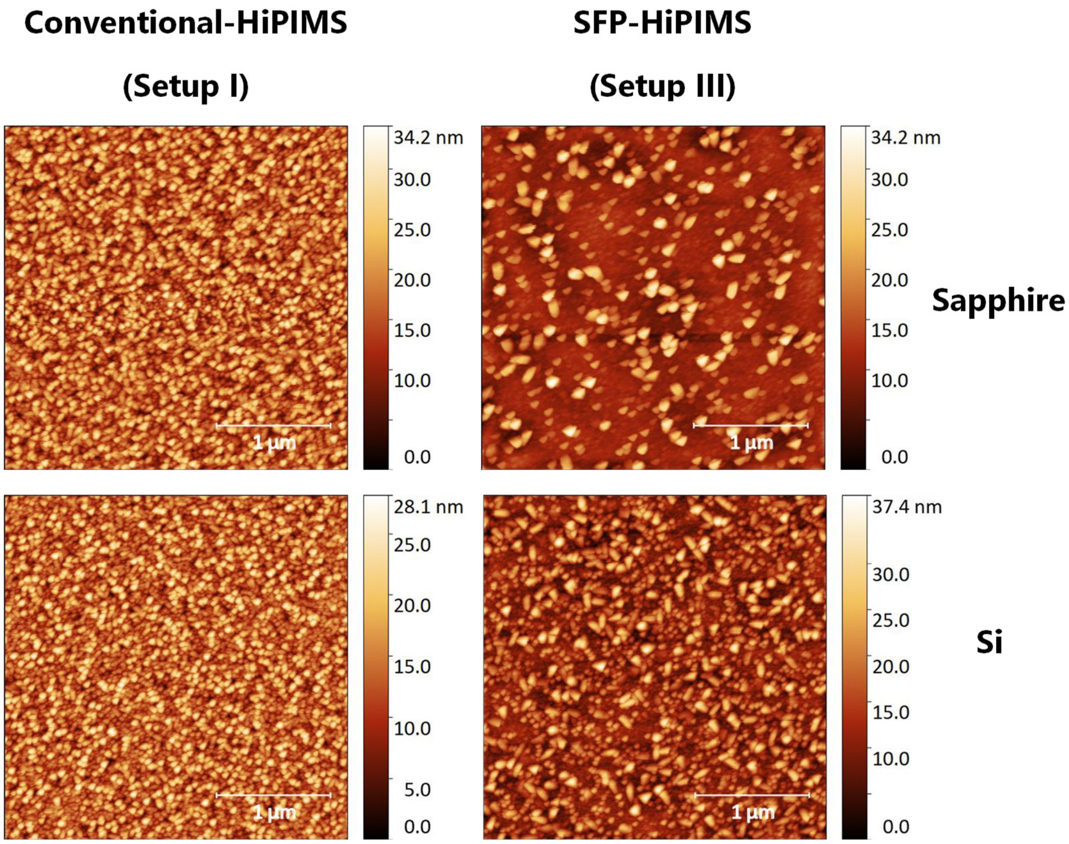

Supplementary Figure 9: AFM images of samples deposited with synchronized setup I and III on sapphire and Si substrate.

The AFM characterization reveals the surface morphology of films deposited using conventional synchronization and SFP-HiPIMS techniques. While the samples have similar thicknesses, the SFP-HiPIMS deposited films display the nucleation of larger grains. This is attributed to enhanced adatom mobility resulting from increased ion bombardment.

Additional AlScN depositions without substrate heating:

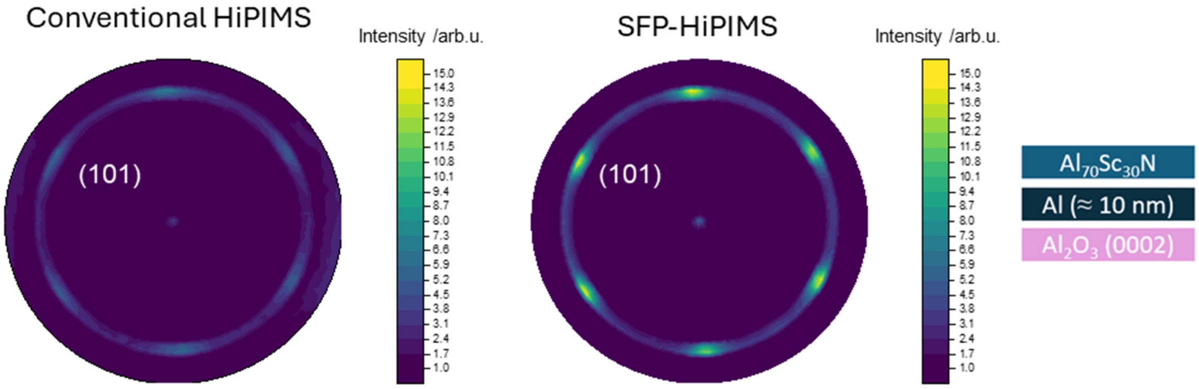

Supplementary Figure 10: Pole figure of the AlScN (101) peak recorded for additional $Al_{70}Sc_{30}N$ samples deposited on Al_2O_3 (0002) using conventional HiPIMS and SFP-HiPIMS.

Finally, to further explore the capabilities of SFP-HiPIMS we performed additional depositions without any intentional substrate heating. The films are deposited using the same general deposition conditions as reported in the main text with slightly higher Sc power and without active substrate heating. An additional 10 nm Al seed layer is introduced between the Al_2O_3 (0002) substrate and the AlScN film. The Al seed layer promotes the c-axis texture during low-temperature growth. Even in this case SFP-HiPIMS offers a marked improvement of the texture and crystalline quality over the conventional process.

Results on SFP-HiPIMS for deposition of denser TaN films

To verify the applicability of SFP-HiPIMS on other material systems, we also deposited TaN films with conventional synchronization (i.e. all pulses start at same time) and SFP-HiPIMS (synchronized with Ta^+ metal ion flux). A minor increase in measured stress values (using wafer curvature) was observed from - 2.26 GPa to - 2.52 GPa with SFP-HiPIMS. However, the ion bombardment led to increased hardness from 14.99±0.97 GPa to 17.83±0.92 GPa, which cannot be caused by the marginal increase in the compressive stresses. Therefore, we assign this improvement in mechanical properties to the densification of the film that is in agreement with previous studies on refractory nitride thin films densified by ion irradiation.[2] The change in the films' properties is less prominent here as compared to AlScN, potentially due to higher lattice displacement threshold of Ta as compared to Al or Sc.[3]

References

[1] A. G. Shard, "Detection limits in XPS for more than 6000 binary systems using Al and Mg Kα X-rays," *Surf. Interface Anal.*, vol. 46, no. 3, pp. 175–185, Mar. 2014, doi: 10.1002/SIA.5406.

[2] A. V. Pshyk, I. Petrov, B. Bakhit, J. Lu, L. Hultman, and G. Greczynski, "Energy-efficient physical vapor deposition of dense and hard Ti-Al-W-N coatings deposited under industrial conditions," *Mater. Des.*, vol. 227, 2023, doi: 10.1016/j.matdes.2023.111753.

[3] A. Y. Konobeyev, U. Fischer, Y. A. Korovin, and S. P. Simakov, "Evaluation of effective threshold displacement energies and other data required for the calculation of advanced atomic displacement cross-sections," *Nucl. Energy Technol.*, vol. 3, no. 3, pp. 169–175, Sep. 2017, doi: 10.1016/j.nucet.2017.08.007.
